# Prevalence, Risk Factors, and Management Strategies of Hyperlipidemia Among Adolescents of a Tertiary Care Rural Indian Setting: A Cross-Sectional Study

**DOI:** 10.7759/cureus.90599

**Published:** 2025-08-20

**Authors:** Danturty L Lalitha, Boddeda Charishma, Nagarjuna Sivaraj, Kiran Veerapaneni, Lakshmi Venkata Simhachalam Kutikuppala

**Affiliations:** 1 Biochemistry, Great Eastern Medical School and Hospital, Srikakulam, IND; 2 Internal Medicine, Great Eastern Medical School and Hospital, Srikakulam, IND; 3 Research and Development, Great Eastern Medical School and Hospital, Srikakulam, IND; 4 Internal Medicine, Dr. M.G. Ramachandran Educational and Research Institute, Chennai, IND; 5 Medicine and Surgery, Dr. NTR University of Health Sciences, Vijayawada, IND

**Keywords:** adolescents, cardiovascular risk, hyperlipidemia, india, rural health

## Abstract

Introduction: Hyperlipidemia is the abnormal blood lipid levels, which are increasingly being recognized in adolescents, a critical stage for establishing lifelong metabolic health. Early dyslipidemia contributes to premature atherosclerosis and cardiovascular disease, major global causes of mortality. In India, rising obesity, unhealthy diets, and reduced physical activity among youth have accelerated this trend. The burden is likely underestimated in rural areas, where screening is rare and awareness is low, allowing many cases to remain undetected until adulthood.

Objectives: To determine the prevalence and identify the demographic, lifestyle, and clinical risk factors associated with hyperlipidemia among adolescents of a rural tertiary care Indian setting in India.

Materials and Methods: A cross-sectional observational study was conducted among students aged 15-19 years. Participants were evaluated for demographic characteristics, anthropometric measurements, lipid profile parameters, lifestyle behaviors, and awareness regarding hyperlipidemia. Statistical analyses were performed to identify significant associations using t-tests and chi-square tests.

Results: Significant gender-based differences were observed in demographic variables, including age, height, and weight, among adolescents with hyperlipidemia (p = 0.000), although BMI was not statistically different between males and females (p = 0.715). Lipid profile variations were seen across academic years and gender. High-density lipoprotein (HDL) and triglycerides showed significant differences in Class-10 students (p = 0.000, p = 0.006), while low-density lipoprotein (LDL) levels became significantly elevated in Class-11 students (p = 0.002). In Class 12, LDL and triglycerides remained significantly associated (p = 0.006, p = 0.000).

Conclusion: Hyperlipidemia in adolescents is influenced by a combination of demographic characteristics, lipid profile abnormalities, lifestyle behaviors, and awareness levels. Early lifestyle interventions, education, and regular screening are warranted to reduce future cardiovascular risk in this vulnerable age group.

## Introduction

Hyperlipidemia, characterized by elevated circulating concentrations of lipids, particularly cholesterol and triglycerides, represents a significant and increasingly prevalent public health concern. This metabolic disorder is a well-established modifiable risk factor for the development of atherosclerosis and subsequent cardiovascular diseases (CVD), including coronary artery disease (CAD), cerebrovascular accidents (stroke), and peripheral arterial disease (PAD) [1]. CVDs remain the leading cause of global morbidity and mortality, accounting for approximately 17.9 million deaths annually, as reported by the World Health Organization (WHO) [2]. The rising incidence of hyperlipidemia has been largely attributed to the widespread adoption of sedentary lifestyles, unhealthy dietary patterns, and the escalating global prevalence of obesity [3]. Furthermore, genetic predisposition and the presence of comorbidities such as diabetes mellitus, hypertension, and metabolic syndrome are known to exacerbate dyslipidemic profiles, thereby compounding cardiovascular risk [4]. Despite notable advancements in pharmacological interventions and the promotion of lifestyle modifications, the effective control of hyperlipidemia remains suboptimal across diverse populations, highlighting the need for ongoing research and the implementation of context-specific, evidence-based strategies [5]. Tertiary care hospitals, which function as referral centers for patients with complex medical conditions, provide a distinct and valuable environment for the comprehensive assessment of hyperlipidemia. These centers manage a wide range of patients with varying severity of dyslipidemia, enabling a thorough evaluation of prevalence patterns and the identification of associated risk factors [6]. Additionally, tertiary care settings offer an opportunity to critically assess the implementation and effectiveness of current management protocols, including pharmacotherapy, lifestyle interventions, and structured patient education programs [7]. Understanding the prevalence and risk profile of hyperlipidemia within tertiary care institutions holds significant clinical and public health relevance. Firstly, such data enable the estimation of disease burden in high-risk cohorts, thereby informing institutional healthcare planning and resource distribution. Secondly, delineation of modifiable and non-modifiable risk factors can facilitate the development of targeted prevention and intervention strategies. Lastly, the systematic evaluation of contemporary treatment practices may reveal gaps in care delivery, thus providing insights to optimize clinical outcomes through evidence-based guideline development [7]. Although traditionally hyperlipidemia has been associated with older populations, recent trends indicate a rise in hyperlipidemia among younger adults due to stress, lifestyle factors, and dietary habits. Understanding the prevalence, risk factors, and management strategies of hyperlipidemia in this demographic is crucial for early intervention and long-term health outcomes [8]. This cross-sectional study was undertaken to estimate the prevalence of hyperlipidemia and to examine its demographic, lifestyle, and clinical correlates among adolescents in a rural tertiary care setting in India.

## Materials and methods

A cross-sectional study was conducted over a two-month period among students aged 15-19 years, targeting adolescents enrolled during the study time frame. A total of 131 participants were selected using a systematic random sampling method, with the sample size calculated based on an estimated prevalence of 13.9%, a 90% confidence level, and a 5% margin of error [9]. The sample size was determined using the standard formula incorporating the z-score, margin of error (ε), population size (N), and estimated population proportion (p̂). The study included adolescents aged 12 to 18 years who were enrolled in any year of schooling or higher secondary education, provided informed consent, and did not have any chronic illnesses or genetic disorders known to directly cause hyperlipidemia, except for those diagnosed with hyperlipidemia as their primary condition. Students were excluded if they were above 18 years of age, declined to provide consent, or had chronic illnesses such as diabetes mellitus, chronic kidney disease, or genetic disorders like familial hypercholesterolemia, which could confound the association with hyperlipidemia. Additionally, students who were pregnant, lactating, undergoing lipid-altering pharmacotherapy (unless prescribed for hyperlipidemia), or who had experienced major medical interventions or surgery within the preceding three months were excluded to minimize bias. Incomplete or missing medical records also led to exclusion. The inclusion and exclusion criteria were designed to focus on a well-defined demographic group, eliminating potential confounders and ensuring that the study primarily assessed hyperlipidemia influenced by modifiable lifestyle and environmental factors, rather than underlying medical conditions or physiological changes unrelated to the study objectives. The data was collected using a structured questionnaire and laboratory tests. Data was collected using a structured, self-designed questionnaire developed by the authors for the purpose of this study. The questionnaire included both closed and open-ended questions covering demographic data (age, gender, year of study), lifestyle factors (dietary habits, physical activity levels, smoking, alcohol consumption), clinical history (family history of hyperlipidemia, personal medical history), awareness and attitudes toward hyperlipidemia and its management, and self-reported management strategies. The questionnaire was prepared in English, translated into the local language (Telugu), and pretested on a subset of 15 adolescents for clarity and comprehension. Internal consistency was assessed using Cronbach’s alpha, which yielded a value of 0.78, indicating acceptable reliability for use in this setting, consistent with values reported in similar Indian adolescent health studies. The final version of the questionnaire is included in the Appendices. Since the tool was self-developed and not previously published, it does not require citation or third-party permission. The questionnaire encompassed demographic data (age, gender, year of study), lifestyle factors (diet, physical activity, smoking, alcohol consumption), clinical history (family history of hyperlipidemia, personal medical history), awareness and attitudes toward hyperlipidemia and its management, and laboratory tests (total cholesterol, low-density lipoprotein (LDL), High-density lipoprotein (HDL), triglycerides) conducted at the college's health center. Serum cholesterol levels were estimated using the modified Roeschlau method, and triglyceride levels were measured by the GPO-Trinder enzymatic method [9-11]. HDL and LDL concentrations were determined using direct enzymatic assays. Ethical approval for the study was obtained from the Institutional Ethics Committee prior to the initiation of data collection with letter no. 191/IEC/GEMS&H/2024. Informed consent was obtained from all participants before their inclusion in the study. Written informed consent was obtained from parents or legal guardians of participants aged below 18 years, and assent was obtained from the adolescents themselves. Participant confidentiality was maintained throughout the research process. Measures were taken to ensure that the identity of participants remained protected throughout the study. Each student was given a code number, and no names or personal details were written on the questionnaires or entered into the main dataset. The list linking codes to names was kept separately in a secure, password-protected file that only the principal investigator could access. Paper forms were stored in a locked cupboard, and electronic records were saved on a computer with restricted access. Results were analyzed and presented in group form so that no individual could be recognized. Even with the demographic information collected, it was not possible to trace responses back to a specific student without the separate code list, which was not used in the analysis.

Statistical analysis

Descriptive statistics were employed to summarize the demographic and clinical characteristics of the study population. The prevalence of hyperlipidemia was calculated to determine its distribution within the cohort. Statistical analyses, including the calculation of mean ± standard deviation (SD), odds ratios (OR), and chi-square tests, were performed to identify risk factors associated with hyperlipidemia. Additionally, the participants’ level of awareness and the management strategies adopted for hyperlipidemia were systematically evaluated. Chi-square tests and cross-tabulation analyses were also performed to assess associations between gender- and class-specific lipid profile differences (HDL, LDL, triglycerides) and the strategies adopted to manage hyperlipidemia, including dietary changes, physical activity, and healthcare consultation.

## Results

A total of 131 adolescents (58 males and 73 females) from government junior colleges of a tertiary care rural setting of South India were studied. The demographic characteristics of the study participants are summarized in Table 1. Statistically significant differences were observed between male and female adolescents with respect to mean age, height, and weight (p = 0.000). However, no significant difference was noted in BMI between the two groups (p = 0.715).

**Table 1:** Demographic Characteristics of Adolescents Diagnosed with Hyperlipidemia Values are presented as mean ± standard deviation (SD). Statistical comparisons were made using an independent samples t-test.

Demographic Parameter	Males (N = 58)	Females (N = 73)	t-value	p-value
Age (years)	15.1 ± 1.6 (mean ± SD)	14.7 ± 1.3 (mean ± SD)	1.436	0.000
Height (cm)	160.8 ± 7.2	151.4 ± 5.8	1.364	0.000
Weight (kg)	50.4 ± 8.6	47.6 ± 7.3	1.287	0.000
Body Mass Index (BMI, kg/m²)	19.6 ± 3.1	20.5 ± 3.4	1.068	0.715

Lipid profile distributions among male and female adolescents diagnosed with hyperlipidemia are presented in Table 2. Among Class-10 students, significant differences were observed between males and females in HDL and triglyceride levels (p = 0.000, p = 0.006, respectively), while LDL and total cholesterol levels did not show statistical significance (p = 0.439, p = 0.505). In Class-11 students, LDL levels were significantly different between genders (p = 0.002), whereas HDL, triglycerides, and cholesterol did not demonstrate significant variation (p = 0.129, p = 0.776, p = 0.382, respectively). In Class-12 students, LDL and triglyceride levels also showed significant gender-based differences (p = 0.006, p = 0.000), while HDL and cholesterol levels remained statistically non-significant (p = 0.147, p = 0.038).

**Table 2:** Lipid Profiles of Adolescents Diagnosed with Hyperlipidemia by Academic Class and Gender All values are presented as mean ± standard deviation (SD). Lipid parameters are expressed in milligrams per deciliter (mg/dL). Statistical comparison between male and female students was performed using an independent samples t-test.

Academic Class	Lipid Parameter (units)	Male (N = 58) (Mean ± SD)	Female (N = 73) (Mean ± SD)	t-value	p-value
Class 10	HDL (mg/dL)	42.54 ± 8.92	48.83 ± 11.18	1.570	0.000
	LDL (mg/dL)	160.35 ± 23.42	164.08 ± 31.64	1.824	0.439
	Triglycerides (mg/dL)	121.42 ± 44.66	101.17 ± 37.67	1.405	0.006
	Total Cholesterol (mg/dL)	90.43 ± 16.70	92.84 ± 24.44	2.141	0.505
Class 11	HDL (mg/dL)	54.33 ± 17.56	50.04 ± 13.68	1.647	0.129
	LDL (mg/dL)	153.64 ± 31.18	168.80 ± 22.88	1.857	0.002
	Triglycerides (mg/dL)	104.43 ± 27.21	106.36 ± 49.34	3.287	0.776
	Total Cholesterol (mg/dL)	88.36 ± 23.72	91.74 ± 19.23	1.520	0.382
Class 12	HDL (mg/dL)	46.14 ± 23.01	50.82 ± 8.77	6.876	0.147
	LDL (mg/dL)	148.71 ± 27.35	161.78 ± 26.50	1.065	0.006
	Triglycerides (mg/dL)	75.43 ± 21.08	95.70 ± 40.24	3.642	0.000
	Total Cholesterol (mg/dL)	96.71 ± 11.97	90.67 ± 20.71	2.991	0.038

A positive family history of hyperlipidemia was more frequently reported among female students, whereas the majority of both male (n = 50, 86.20%) and female (n = 63, 86.30%) students reported no history of smoking, alcohol consumption, or pharmacological management for hyperlipidemia. The clinical history of comorbid conditions among adolescents is detailed in Table 3. The presence of obesity showed conditional associations with hyperlipidemia in males (n = 3, 5.2%) and females (n = 5, 6.8%). Thyroid disorders were reported more frequently among females (n = 1, 1.37%) than males (n = 0, 0.0%). Hypertension and CVDs were not significantly associated with hyperlipidemia in this cohort. The majority of both male (n = 50, 86.20%) and female (n = 63, 86.30%) students did not exhibit clinical comorbidities associated with hyperlipidemia.

**Table 3:** Clinical History of Adolescents Diagnosed with Hyperlipidemia

Medical Condition	Males (N = 58)	Females (N = 73)
Hypertension	0 (0.00%)	0 (0.00%)
Diabetes mellitus	0 (0.00%)	0 (0.00%)
Obesity	3 (5.17%)	5 (6.85%)
Thyroid disorders	0 (0.00%)	1 (1.37%)
Cardiovascular diseases	0 (0.00%)	0 (0.00%)
None	50 (86.20%)	63 (86.30%)
Others (unspecified)	2 (3.45%)	2 (2.74%)

Table 4 represents lifestyle habits associated with hyperlipidemia risk. No statistically significant differences were observed between males and females in the frequency of fruit and vegetable intake, fast-food consumption, or intake of fried foods and sugary beverages, with most students reporting moderate weekly consumption in all categories.

**Table 4:** Daily Lifestyle Modifications Affecting Hyperlipidemia in Adolescents

Lifestyle Behavior	Males (N = 58)	Females (N = 73)	Chi-square	p-value
Fruit and Vegetable Consumption				
Daily	16 (27.58%)	17 (23.28%)	0.328	0.954
3–4 days/week	18 (31.03%)	21 (28.76%)		
1–2 days/week	20 (34.48%)	25 (34.24%)		
Rare/Never	6 (10.34%)	9 (12.33%)		
Fast-Food Consumption				
Daily	4 (6.89%)	1 (1.37%)	2.618	0.454
3–4 days/week	13 (22.41%)	15 (20.55%)		
1–2 days/week	31 (53.44%)	41 (56.16%)		
Rare/Never	12 (20.68%)	13 (17.80%)		
Fried Food Consumption				
Daily	3 (5.17%)	3 (4.11%)	0.954	0.812
3–4 days/week	14 (24.13%)	13 (17.81%)		
1–2 days/week	32 (55.17%)	44 (60.27%)		
Rare/Never	9 (15.51%)	13 (17.81%)		
Sweets and Sugary Beverage Intake				
Daily	1 (1.72%)	8 (10.95%)	9.012	0.029
3–4 days/week	13 (22.41%)	17 (23.28%)		
1–2 days/week	37 (63.79%)	30 (41.09%)		
Rare/Never	7 (12.06%)	16 (21.92%)		
Physical Activity Frequency				
Daily	19 (32.75%)	10 (13.69%)	12.430	0.006
3–4 days/week	13 (22.41%)	7 (9.58%)		
1–2 days/week	17 (29.31%)	35 (47.94%)		
Rare/Never	9 (15.51%)	17 (23.28%)		

Table 5 presents the participants’ knowledge and awareness of hyperlipidemia. Overall, both male and female students demonstrated high awareness of the condition, normal cholesterol ranges, and relevant dietary recommendations, with no statistically significant differences between the groups. The prevalence ratio (PR) values in Table 5 are close to 1, which indicates minimal difference in knowledge and awareness between male and female participants. These values represent the relative likelihood of awareness between the two groups and do not imply any causal relationship between knowledge levels and the presence of hyperlipidemia.

**Table 5:** Knowledge and Awareness of Hyperlipidemia Among Adolescents *Odds Ratio confidence intervals were calculated using the Woolf logit method. Odds Ratio (OR) and Prevalence Ratio (PR) values in this table compare knowledge and awareness between male and female participants. They indicate the relative likelihood of awareness across sexes and should not be interpreted as measures of association between knowledge levels and the presence of hyperlipidemia.

Knowledge and Awareness Domain	Males (N = 58)	Females (N = 73)	Odds Ratio (95% CI)	PR
Awareness of Hyperlipidemia				
Yes	53 (91.38%)	67 (91.78%)	0.9493 (0.2275, 4.163*)	0.995
No	5 (8.62%)	6 (8.22%)		
Knowledge of Normal Cholesterol Values				
Yes	53 (91.38%)	68 (93.15%)	0.7794 (0.1701, 3.584*)	0.981
No	5 (8.62%)	5 (6.85%)		
Knowledge of Dietary Recommendations to Manage/Prevent Hyperlipidemia				
Yes	47 (88.67%)	61 (83.56%)	0.8405 (0.309, 2.310*)	0.969
No	11 (18.96%)	12 (16.43%)		

Management strategies employed by the students are detailed in Table 6. Male students diagnosed with hyperlipidemia were more likely to implement dietary modifications compared to females. A substantial proportion of both males (n = 21, 36.20%) and females (n = 21, 28.76%) reported not consulting healthcare professionals regarding lipid profile management. Among those who did seek medical advice, follow-up patterns varied, with annual check-ups being more common among female students (n = 12, 16.43%). Chi-square analysis revealed that students who adopted at least one active management strategy - such as regular physical activity or dietary modification - tended to have more favorable HDL and triglyceride profiles, particularly among Class-11 and Class-12 students (p < 0.05). These associations also showed gender-based differences, with females demonstrating better lipid outcomes when combining dietary adherence with regular healthcare follow-up.

**Table 6:** Management Strategies of Hyperlipidemia in Adolescents

Questioning for Awareness of Hyperlipidemia	Males N=58 (%)	Females N=73 (%)
If you have been diagnosed with hyperlipidemia, what management strategies have you adopted?		
Dietary modifications	42 (72.41%)	26 (35.61%)
Regular exercise	7 (12.06%)	8 (10.95%)
Medication	0 (0%)	0 (0%)
Regular monitoring of lipid levels	5 (8.62%)	16 (21.91%)
None	14 (24.13%)	23 (31.50%)
How often do you follow up with a healthcare professional regarding your lipid levels?		
Monthly	8 (13.79%)	5 (6.84%)
Every 3 months	6 (10.34%)	9 (12.32%)
Every 6 months	6 (10.34%)	8 (10.95%)
Annually	8 (13.79%)	12 (16.43%)
Often when I feel unwell	9 (15.51%)	18 (24.65%)
Never	21 (36.20%)	21 (28.76%)
On a scale of 1-5, how compliant are you with your prescribed management plan?		
1	20 (34.48%)	17 (23.28%)
2	7 (12.06%)	6 (8.21%)
3	19 (32.75%)	32 (43.83%)
4	4 (6.89%)	12 (16.43%)
5 (highly compliant)	8 (13.79%)	6 (8.21%)

Perceived barriers and facilitators to hyperlipidemia management are outlined in Table 7. Lack of time was identified as the most commonly reported barrier by both males (n = 28, 48.27%) and females (n = 39, 53.42%). Additional barriers included insufficient knowledge (females (n = 39, 53.42%) vs. males (n = 9, 15.06%)), financial constraints, lack of motivation, and other personal factors.

**Table 7:** Perceived Barriers and Facilitators to Manage the Hyperlipidemia

Barriers to Management	Males N=58 (%)	Females N=73 (%)
Lack of time	28 (48.27%)	39 (53.42%)
Others	10 (17.24%)	8 (10.95%)
Lack of concentration	6 (10.34%)	11 (15.06%)
Lack of knowledge	5 (8.62%)	11 (15.06%)
Financial constraints	2 (3.44%)	1 (1.36%)
Side effects of medication	0 (0%)	2 (2.73%)
Facilitators to Better Management		
More information and education	39 (67.24%)	47 (64.38%)
Better access to healthcare services	9 (15.51%)	13 (17.80%)
Other	8 (13.79%)	8 (10.95%)
Support from family and friends	2 (3.44%)	5 (6.84%)
Financial assistance	0 (0%)	0 (0%)

## Discussion

Prevalence and demographic differences

The present study evaluated the prevalence and determinants of hyperlipidemia among adolescents enrolled in junior colleges, with emphasis on gender-based differences. Significant variations were observed in demographic parameters such as age, height, and weight between male and female students with hyperlipidemia (p = 0.000), while BMI did not differ significantly (p = 0.715). This suggests that BMI alone may be an inadequate predictor of hyperlipidemia risk in adolescents, highlighting the need for comprehensive metabolic screening rather than reliance on anthropometric measures alone.

Lipid profile variations across academic years

Gender-related differences in lipid metabolism were evident and appeared to evolve with academic stage. In Class-10 students, HDL and triglycerides were significantly different between males and females (p = 0.000 and p = 0.006, respectively), while LDL and total cholesterol were similar. Class-11 students showed a significant gender difference in LDL levels (p = 0.002), and Class-12 students demonstrated significant variation in both LDL-C and triglycerides (p = 0.006 and p = 0.000, respectively). These results imply that age-related metabolic changes, academic stress, and lifestyle adaptations during the junior college years may influence lipid profiles.

Lifestyle behaviors and dietary patterns

Dietary habits emerged as key modulators of lipid status. Both genders reported moderate fruit and vegetable consumption (weekly intake by ~34% of both groups), but high consumption of fast and fried foods, sweets, and sugar - sweetened beverages was common - especially among males (63.8%). Physical activity levels varied but were insufficient to offset the effects of unhealthy dietary practices. Chi-square analysis confirmed a significant association between adverse dietary habits and hyperlipidemia, consistent with earlier reports linking dietary excess and sedentary behavior to dyslipidemia in adolescents [12-16].

Management strategies and their impact

Active lifestyle modifications - such as increased physical activity, healthier diets, and healthcare consultations - were associated with more favorable HDL and triglyceride levels, particularly among Class-11 and Class-12 students. Despite this, barriers to effective management included a lack of time, limited knowledge, and, to a lesser extent, financial constraints. Females more frequently cited a lack of knowledge (53.42%) compared to males (15.06%), suggesting gender-specific educational needs.

Family history and awareness levels

A positive family history of hyperlipidemia was more frequent among females, suggesting a possible genetic predisposition and need for earlier screening. Awareness about hyperlipidemia, normal cholesterol ranges, and dietary recommendations was high in both genders (>91%). However, gaps were noted in healthcare-seeking behavior, particularly among males (36.2%) and unhealthy females (24.6%), indicating a disconnect between knowledge and practice.

Comparison with existing literature

The findings align with Zhang et al., who reported an age-related increase in dyslipidemia prevalence, with males typically exhibiting higher serum lipid levels [12]. Similar to prior studies, hypertriglyceridemia was the most common dyslipidemia subtype in males, supporting the role of triglyceride-rich lipoproteins in early atherogenesis [13-19]. Our data also corroborate Al-Shehri et al.’s observation of lower HDL and higher triglycerides in males and parallel trends seen in Bangladeshi adolescents by Ali et al. [20-21]. Evidence from randomized controlled trials confirms that Mediterranean-style diets can significantly reduce triglycerides, LDL, and total cholesterol while improving vascular health, and that regular exercise enhances HDL while lowering LDL and triglycerides [22-31].

The present study highlights the influence of fast-food consumption, excessive intake of sweets and sugar-sweetened beverages, and irregular physical activity on hyperlipidemia prevalence among adolescents, findings that are in agreement with prior research by Mainieri et al. [32]. In this study, knowledge and awareness were measured using simple “Yes/No” questions. Such a format carries the risk of overestimating participants’ understanding, as some may have responded “Yes” despite holding only partial or incorrect information about hyperlipidemia, cholesterol levels, or dietary measures. Allowing respondents to give open-ended answers would have provided a clearer picture of their actual knowledge. Future research could address this by using open-response questions or structured knowledge assessments to more accurately distinguish genuine understanding from misconceptions. The results emphasize the importance of early interventions in lifestyle and dietary habits, along with regular lipid profile monitoring, particularly for students with a family history of hyperlipidemia. Efforts should focus on increasing awareness and accessibility to health care services while addressing the perceived barriers. Universities could incorporate structured wellness programs, provide nutritional counseling, and foster environments that encourage healthy lifestyles to mitigate the long-term risks of hyperlipidemia. This study highlights gender-specific patterns in hyperlipidemia among adolescents, shaped by demographic, clinical, and lifestyle factors. Future research should aim to explore these trends longitudinally and assess the impact of tailored interventions on mitigating the risk of hyperlipidemia in the adolescent phase itself.

Limitations

The study has certain limitations, such as the fact that it was conducted with a relatively small number of participants, which may not fully represent the wider adolescent population. Information was gathered through self-reported questionnaires, which can be influenced by memory errors or the tendency to give socially desirable answers, particularly in items related to lifestyle and knowledge. As this was a cross-sectional study, it provides only a snapshot in time and cannot establish cause-and-effect relationships. The lack of follow-up data also prevents assessment of how lipid profiles or related behaviors may change over time. Furthermore, the recruitment of students from a limited number of institutions may have introduced selection bias, as adolescents from other educational or community settings might have different risk profiles.

## Conclusions

The study highlights a notable prevalence of hyperlipidemia among adolescents, with significant gender-specific clinical associations - such as consistently higher triglyceride levels and lower HDL-C levels observed in males, and a higher frequency of positive family history and insufficient knowledge reported in females. Lifestyle factors, including frequent sweet and beverage consumption and irregular physical activity, emerged as significant contributors across both genders. Despite high levels of awareness regarding hyperlipidemia and dietary guidelines, females reported a need for more knowledge, while males demonstrated lower health-seeking behavior, indicating a disparity in the practical application of health knowledge. These gender differences were also reflected in class-wise lipid variability, with senior students showing more pronounced lipid abnormalities. Targeted interventions, including gender- and class-specific awareness campaigns, regular lipid screening, and structured wellness programs, are essential to mitigate cardiovascular risk and improve long-term metabolic outcomes in this vulnerable population.

## Appendices

Questionnaire for prevalence, risk factors, and management strategies of hyperlipidemia in MBBS students: A cross-sectional study

Section A: Demographic Information

1. Age: _______

2. Gender:

  o Male

  o Female

  o Other

3. Year of study:

   o Class 10

   o Class 11

    o Class 12

4. Height (cm): _______

5. Weight (kg): _______

6. Body Mass Index (BMI): _______ (calculated automatically)

Section B: Lifestyle Factors

7. How often do you consume the following types of food? (Mark the appropriate option)

   o Fruits and Vegetables

      §  Daily

      §  3-4 times a week

      §  1-2 times a week

      §  Rarely/Never

  o Fast Food

     §  Daily

     §  3-4 times a week

     §  1-2 times a week

     §  Rarely/Never

  o   Fried Food

     §  Daily

     §  3-4 times a week

     §  1-2 times a week

     §  Rarely/Never

  o Sweets and Sugary Beverages

    § Daily

    §  3-4 times a week

    §  1-2 times a week

    §  Rarely/Never

8. How often do you engage in physical activity? (e.g., sports, gym, running, etc.)

   o Daily

   o 3-4 times a week

   o 1-2 times a week

   o Rarely/Never

9. Do you smoke?

  o Yes

  o No

10. Do you consume alcohol?

  o Yes

  o No

Section C: Clinical History

11. Do you have a family history of hyperlipidemia?

   o Yes

   o No

   o Don't know

12. Do you have any of the following medical conditions? (Check all that apply)

   o Hypertension

   o Diabetes Mellitus

   o Obesity

   o Thyroid Disorders

   o Cardiovascular Diseases

   o None

   o Other (please specify): _______

13. Are you currently taking any medication for hyperlipidemia?

   o Yes

   o No

Section D: Knowledge and Awareness

14. Are you aware of what hyperlipidemia is?

  o Yes

  o No

15. Do you know the normal range of cholesterol levels?

  o Yes

  o No

16.     Do you know the dietary recommendations to manage or prevent hyperlipidemia?

  o Yes

  o No

Section E: Laboratory Results (To Be Filled by the Medical Team)

17. Total Cholesterol: _______ mg/dL

18.  LDL Cholesterol: _______ mg/dL

19.  HDL Cholesterol: _______ mg/dL

20.  Triglycerides: _______ mg/dL

Section F: Management Strategies

21. If you have been diagnosed with hyperlipidemia, what management strategies have you adopted? (Check all that apply)

o   Dietary modifications

o   Regular exercise

o   Medication

o   Regular monitoring of lipid levels

o   None

o   Other (please specify): _______

22.     How often do you follow up with a healthcare professional regarding your lipid levels?

o   Monthly

o   Every 3 months

o   Every 6 months

o   Annually

o   Only when I feel unwell

o   Never

23. On a scale of 1 to 5, how compliant are you with your prescribed management plan?

o   1 (Not compliant at all)

o   2

o   3

o   4

o   5 (Highly compliant)

Section G: Perceived Barriers and Facilitators

24. What are the main barriers to managing your lipid levels effectively? (Check all that apply)

o   Lack of time

o   Lack of knowledge

o   Financial constraints

o   Lack of motivation

o   Side effects of medication

o   Other (please specify): _______

25. What factors would help you better manage your lipid levels? (Check all that apply)

    o More information and education

    o Better access to healthcare services

    o Support from family and friends

    o Financial assistance

    o Other (please specify): _______

Section H: General Comments

Any additional comments or suggestions regarding the management of hyperlipidemia: _______
